# Contractile Reserve in Heart Failure with Preserved Ejection Fraction

**DOI:** 10.3390/jcdd9080248

**Published:** 2022-08-04

**Authors:** Daniela Di Lisi, Quirino Ciampi, Cristina Madaudo, Girolamo Manno, Francesca Macaione, Salvatore Novo, Giuseppina Novo

**Affiliations:** 1 Department of Health Promotion Sciences, Maternal-Infant Care, Internal Medicine and Specialities of Excellence “G. D’Alessandro”, University of Palermo, Palermo, via del Vespro 129, 90127 Palermo, Italy; 2 Cardiology Unit, University Hospital “P. Giaccone”, Palermo, via del Vespro 129, 90127 Palermo, Italy; 3 Department of Cardiology, Ospedale Fatebenefratelli, 80123 Benevento, Italy

**Keywords:** contractile reserve, echo stress, global longitudinal reserve, heart failure

## Abstract

**Background:** Diastolic stress echocardiography (SE) is useful for confirming the diagnosis of heart failure with preserved left ventricular ejection fraction (HFpEF) when it is uncertain. The aim of this study was to assess the value of new echocardiographic parameters during diastolic SE in patients with dyspnea and suspected HFpEF. **Methods:** Sixty-two patients with exertional dyspnea and inconclusive rest echocardiography for a diagnosis of HFpEF were enrolled. Exercise SE was performed in all patients. Contractile reserve (LVCR) was assessed by measuring: 1. changes in the left ventricular ejection fraction (LVEF) between rest and peak stress; 2. stress-to-rest ratio of force (force was defined as the ratio between systolic arterial pressure and left ventricular end-systolic volume); and 3. mechanical reserve, defined as the change in systolic strain (GLS) between rest and peak stress. **Results:** Diagnosis of HFpEF was performed by SE in 26 patients. Comparing patients with a diagnosis of HFpEF (group A) to patients with other causes of dyspnea (group B), we found a significant increase in the E/e’ ratio in group A at peak stress. LV GLS was significantly reduced in group A compared to group B at rest and stress (*p* value 0.01 at rest; *p* value 0.04 at stress). At peak stress, GLS did not significantly increase in group A, while it increased in group B (*p* value 0.04). LVEF increased significantly in both groups. **Conclusion:** Patients with HFpEF have impaired LVCR when assessed using GLS. Thus, the assessment of mechanical reserve could give additional diagnostic information during stress tests in patients with HFpEF.

## 1. Introduction

Heart failure (HF) is a clinical syndrome characterized by typical symptoms (e.g., breathlessness, ankle swelling and fatigue) that may be accompanied by signs (e.g., elevated jugular venous pressure, pulmonary crackles and peripheral edema) caused by a structural and/or functional cardiac abnormality, resulting in reduced cardiac output and/or elevated intracardiac pressures at rest or during stress.

HF with preserved left ventricular ejection fraction (LVEF)—HFpEF—is diagnosed in patients with preserved LVEF (typically considered LVEF ≥ 50%) [1].

In several clinical trials, patients with an LVEF between 40 and 49% were often classified as HFpEF. The diagnosis of HFpEF remains challenging, and it is based on the presence of signs and symptoms of HF, preserved LVEF, elevated levels of natriuretic peptides (BNP > 35 pg/mL and/or NT-proBNP > 125 pg/mL) and at least one additional criterion (relevant structural heart disease such as left ventricular hypertrophy and/or left atrial enlargement, diastolic dysfunction). In particular, to facilitate the diagnosis of HFpEF, recently, several diagnostic algorithms, such as the HFA–PEFF score, have been identified [2]. The HFA–PEFF score uses functional, morphological and biomarker domains. Within each domain, a major criterion scores 2 points, and a minor criterion scores 1 point. A total score ≥ 5 points is considered to be diagnostic for HFpEF, while a score of ≤1 point is considered to make a diagnosis of HFpEF very unlikely and to mandate investigations for alternative causes. Patients with an intermediate score (2–4 points) need further evaluation, such as a diastolic stress test or invasive hemodynamic measurements [2,3].

The diastolic stress test is very useful in the diagnosis of HFpEF when baseline echocardiogram is not diriment; it entails assessing left ventricle (LV) filling pressures through E/e’ ratio measurement and tricuspid regurgitation (TR) peak velocity, which indicates systolic pulmonary artery pressure (SPAP), during stress. It is known that an average E/e’ ratio ≥ 15 at rest has high diagnostic value in identifying high pulmonary capillary wedge pressure (PCWP). An E/e’ ratio within the intermediate range is less sensitive [4,5]. Exercise echocardiography should be considered abnormal if the average E/e’ ratio at peak stress increases to ≥15, with or without a peak TR velocity > 3.4 m/s [6]. When a diagnosis of HFpEF is uncertain, invasive hemodynamic measurements with the assessment of filling pressures (PCWP ≥ 15 mmHg) or left ventricular end-diastolic pressure (LVEDP ≥ 16 mmHg) remain the gold-standard diagnostic technique. Hemodynamic measurements following exercise can also be performed, with the assessment of changes in filling pressures, SPAP, stroke volume and cardiac output, if rest values remain below thresholds [7].

In the HFA–PEFF score, left ventricle global longitudinal strain (GLS) < 16% is a minor criterion for a diagnosis of HFpEF.

Recently, several studies assessed the role of global left ventricular contractile reserve (LVCR) in different diseases, such as severe mitral regurgitation and myocardial ischemia [8]. Contractile reserve can be assessed as the absolute change and percent change in LVEF during stress echo; as the stress-to-rest ratio of force; and as mechanical reserve (MRES), defined as the percent change in systolic strain at the stress peak or immediately post-exercise.

Force (or elastance) is the ratio between systolic arterial pressure measured by cuff sphygmomanometer and end-systolic volume determined by two-dimensional echocardiography.

Force seems to have prognostic value in ischemic and non-ischemic diseases. In both ischemic and non-ischemic hearts, preserved force is associated with a more benign prognosis [9].

Additionally, preserved LVCR, assessed by speckle tracking echocardiography, was associated with better results after percutaneous edge-to-edge mitral valve repair (PMVR) in the setting of advanced HF; it was associated with better outcomes [8].

LCVR was also assessed in HFpEF patients, particularly as changes in LVEF or elastance.

However, to date, mechanical reserve has not been analyzed in HFpEF. Thus, the purpose of the present study was to identify new rest and stress echocardiographic diagnostic parameters in patients with dyspnea and suspected HFpEF. In particular, our study aimed to investigate the role of GLS and LVCR (specifically assessed using speckle tracking echocardiography—STE) in patients with HFpEF.

## 2. Methods

Patients referred to our hospital to perform a stress echo for exertional dyspnea and inconclusive rest echocardiography for a diagnosis of HFpEF were enrolled from March 2018 to October 2019. Inclusion criteria were: inexplicable exertional dyspnea, preserved ejection fraction (LVEF ≥ 40%), normal left ventricular filling pressures estimated by the E/e’ ratio (average E/e’ ratio ≤ 14) at rest, good exercise capacity and age between 18 and 85 years old.

Exclusion criteria were: grade > 1 diastolic dysfunction at rest, prior history of myocardial infarction and/or previous myocardial revascularization, significant heart valve diseases or inadequate acoustic window to perform analysis of contractile reserve.

Cardiological evaluation, including clinical examination, 12-lead electrocardiogram and 2D transthoracic echocardiogram with speckle tracking echocardiography at rest and stress, was performed in all patients. The HFA–PEFF score was calculated in all patients during clinical examination [2]. All patients had an HFA–PEFF score between 2 and 4.

Transthoracic echocardiography was performed using a commercially available ultrasound system (GE Vivid E95 ultrasound, GE Healthcare, Horten, Norway) with a variable-frequency phased-array transducer (2.5 MHz). All patients underwent semi-supine bicycle stress echo after the resting echocardiogram using a standard protocol (incremental steps of 25 Watts for 3 min). Systolic blood pressure was taken at baseline and at the end of each stage. A 12-lead ECG was recorded at rest and at the end of each stage.

The end points of the study included: new wall motion abnormalities, significant ST segment changes, significant symptoms or arrhythmias or conclusion of the protocol.

On the basis of currently accepted criteria, the test was considered positive for diastolic dysfunction when all of the following three conditions were met during exercise: exercise average E/e’ ratio > 14 or septal E/e’ ratio > 15, exercise peak tricuspid regurgitant jet velocity > 2.8 m/s, and rest septal e’ velocity < 7 cm/sec or lateral e’ velocity < 10 cm/sec [10,11].

In particular, using the HFA-PEFF score, an average E/e’ ratio during exercise ≥15 added 2 points to the HFA–PEFF score; an average E/e’ ratio ≥ 15 with a peak TR velocity > 3.4 m/s added 3 points to the previous score, including points for functional, morphological and biomarker evaluation. If the combined score was ≥5 points, then the diagnosis of HFpEF was confirmed [2]. Echocardiographic parameters measured at baseline and at peak effort included: LVEF, GLS, left atrial volume (LA), E/e’ ratio, tricuspid annular plane excursion (TAPSE), systolic pulmonary artery pressure (SPAP) and force.

All echocardiographic examinations were performed according to the Recommendations for Cardiac Chamber Quantification by Echocardiography in Adults of the American Society of Echocardiography and the European Association of Cardiovascular Imaging [12]. LVEF was derived by measuring LV end-diastolic and end-systolic volumes with the Simpson biplane method. LA was indexed for body surface area (LAVi), and it was measured in 4 and 2 chamber apical views. SPAP was derived from the tricuspid regurgitation velocity gradient and inferior vena cava size and reactivity. The average E/e’ ratio was obtained by measuring early diastolic myocardial velocity (E’) of basal septal and basal lateral walls with pulsed tissue Doppler imaging and E wave with pulsed Doppler imaging of transmitral flow.

LV GLS was assessed using speckle tracking echocardiography.

Apical four-, two- and long-axis views were acquired with a frame rate between 50 and 80 fps for three consecutive heart cycles. EchoPAC Software version 112.0.0 (GE Healthcare, Chicago, IL, USA) Vingmed Ultrasound was used to analyze the recorded digital cine-loop images and to quantify GLS.

Tracking quality was assessed by the operator and scored by the software by the automated function in the region of interest, which was adjusted by correcting the endocardial border or width if deemed necessary. Aortic valve closure was identified using the automated function from the apical long-axis view. GLS was calculated by averaging the local strains of all 17 segments and expressed as a bull’s eye. We also assessed the reproducibility of GLS measurements between two different expert operators (blind analysis). Intra- and interobserver correlation for GLS (for rest and peak stress values) was 97% and 94%, respectively.

LV contractile reserve (LVCR) was assessed as: 1. percent LVEF change; 2. the stress-to-rest ratio of force; and 3. percent change in systolic strain at the stress peak (mechanical reserve: MRES). Force, also known as elastance, was expressed as the ratio of peak systolic blood pressure measured by cuff sphygmomanometer and end-systolic volume (ESV) at 2D echocardiography.

According to the current recommendations, preserved LVCR was defined as an increase in LVEF ≥ 5% at peak stress from rest and/or an increase in GLS ≥ 2% [13].

In addition, the cut-off value for preserved LVCR assessed using force was considered > 2.0 for exercise stress [14,15].

## 3. Statistical Analysis

Statistical analyses were performed using MedCalc 12.6.0 (MedCalc Software, Mariakerke, Belgium) and PSS for Mac, release 18.0 (Chicago, IL, USA). Continuous variables were expressed as mean ± standard deviation (SD) or, when the data were not normally distributed, as median with the interquartile range and the confidence interval (CI); categorical variables were expressed as percentages.

When the data were normally distributed, we used Student’s t-test to compare groups, and when they were not, we used the Mann–Whitney U test. The Chi Square test was used to compare categorical variables. A *p* value < 0.05 was considered statistically significant.

Independent predictors of HFpEF were assessed by multivariable logistic regression analysis. Odds ratios with the corresponding 95% confidence intervals were estimated.

The selection of independent predictors was performed for a logistic hazards regression model using a *p* value of 0.1 as the threshold for inclusion in the model. A probability value of <0.05 was considered statistically significant.

## 4. Results

Among patients referred to our echo lab for inexplicable dyspnea during the recruitment period, we enrolled 62 patients, of whom 26 were diagnosed as having HFpEF after performing exercise stress echocardiography (group A). All 26 patients had an HFA-PEFF score ≥ 5 points. The remaining patients were considered to have non-cardiogenic dyspnea (group B).

Patients’ characteristics are summarized in Table 1.

The mean age of the general population was 62± 10.6, with a similar prevalence of men and women (50% vs. 50%). Considering cardiovascular risk factors, 83% of patients were hypertensive and were treated with beta-blockers (48%), angiotensin-converting enzyme (ACE) inhibitors (42%) or angiotensin receptor blockers (ARBs) (27%); 51% of patients had dyslipidemia, and 38% had diabetes; 20% of patients were smokers. 

The mean value of BMI was 27 ± 3, indicating a rather overweight study population.

At rest, all patients had normal values of LVEF (LVEF 60 ± 6%) and normal left ventricular filling pressures (average E/e’ ratio 7.7 ± 2.4). The mean GLS value was −18.7% ± 3.3 (normal reference values: −19.7%, CI 95% from −20.4% to 18.9%) [12].

No statistically significant differences were found regarding the general characteristics of patients between group A and group B (Table 1).

In particular, according to the literature, we found that HFpEF was more prevalent in women [16].

A greater percentage of smokers were present in group B.

Arterial hypertension was more prevalent in patients with HFpEF (group A).

Comparing echocardiographic data between the two groups, we found that at rest and stress, patients with HFpEF had statistically significantly higher values of SPAP compared to group B (*p* value 0.01 at rest; *p* value < 0.0001 at stress) and LAVi (*p* value 0.03 at rest and *p* value < 0.0001 at stress) and lower values of GLS (*p* value 0.01 at rest; *p* value 0.04 at stress) compared to group B.

The average E/e’ was significantly increased in group A compared to group B at rest (*p* value 0.001) and stress (*p* value < 0.0001).

At peak exercise, we observed a significant increase in SPAP in both groups (*p* value < 0.0001 in group A and *p* value < 0.0001 in B group). LAVi did not increase significantly in either group at peak exercise. A significant increase in the average E/e’ ratio at peak exercise was present in group A (*p* value 0.0001) and not in group B (*p* value 0.27). GLS did not significantly increase with stress in group A (*p* value 0.58), while it increased in group B (*p* value 0.04). LVEF significantly increased in both groups. Force increased significantly at stress in both groups (*p* value 0.001 in group A and *p* value < 0.0001 in group B). The heart rate, systolic arterial pressure (SAP) and diastolic arterial pressure (DAP) also significantly increased in both groups (Table 2).

The impairment of LVCR measured by GLS was more frequent in patients with HFpEF compared to patients with other causes of dyspnea, while no differences were found regarding LVCR measured by changes in LVEF and force. In particular, mechanical reserve was reduced in all patients of group A and preserved in group B in 14% of patients. This difference between group A and group B was statistically significant (*p* value 0.04). When assessing LVCR through the percentage increase in LVEF, we did not find significant differences between groups. Only 46% of the patients in group A had changes in LVEF ≥ 5% at stress vs. 53 % of patients in group B (*p* value 0.5). LCVR measured through the ratio of force peak/force rest was reduced in both groups (1.6 in group A and 1.7 in group B, *p* value = 0.4). In particular, in group A, 19% had normal LVCR-force vs. 42% in group B (*p* value 0.06) (Table 3).

In addition, a multivariable logistic regression analysis was performed, comparing GLS, LVEF and force at rest, at peak and the difference between these two values (Δ peak-rest). For HFpEF, only GLS variables were associated with an increased likelihood of accuracy in the diagnosis (Table 4).

## 5. Discussion

HFpEF is a challenging diagnosis. Several scores, such as the HFA-PEFF score, have been created to facilitate this diagnosis.^2^ Moreover, stress echocardiography can be of aid in patients with an inconclusive rest echocardiogram. If the HFA–PEFF score remains <5 points or if exercise echocardiography cannot be performed, an invasive hemodynamic stress test is recommended in cases of doubt, especially if the results will drive therapeutic decisions [2].

In our study, new echocardiographic parameters were tested during exercise stress tests, together with conventional ones, to diagnose HFpEF in patients with exertional dyspnea.

According to the literature data, in our study, patients with HFpEF were predominantly female and hypertensive [17].

Conversely, smoking was more prevalent in patients with other causes of dyspnea.

According to the literature, patients with HFpEF and with an HFA–PEFF score < 5, compared to patients with other causes of dyspnea, had a significant increase in the E/E’ ratio at peak exercise and a significant increase in SPAP. The increase in TR velocity might be caused simply by a normal hyperdynamic response to exercise in the absence of LV diastolic dysfunction, and it should not be used to diagnose HFpEF without an E/E’ ratio increase [18].

Certainly, SPAP measurement during exercise can be helpful in aiding the assessment of diastolic filling pressure [19]. It has been shown that the upper normal limit of SPAP is 35 mmHg at rest and 43 mmHg during exercise [20].

In addition, in our study, we found that patients with HFpEF had reduced values of GLS not only at baseline but also during stress. These data are in agreement with the literature. In fact, existing data show that left ventricular deformation is altered despite preserved LVEF in conditions predisposing to heart failure (HF), including increasing age, hypertension, diabetes, renal dysfunction and obesity [21,22,23,24].

LV deformation assessed by speckle tracking echocardiography (STE) has been proven to detect subclinical cardiac involvement and have prognostic value in different pathologic conditions [25]. Furthermore, GLS has been demonstrated to predict 5-year all-cause mortality risk in patients with acute heart failure independently of LVEF values [26]. In patients with dyspnea and preserved LVEF, left ventricular deformation at rest (especially low longitudinal strain and high circumferential strain) predicts the exercise-induced rise in pulmonary artery wedge pressure (PAWP) [27].

A study showed that, in patients with HFpEF, both longitudinal strain (LS) and circumferential strain were impaired, and LS in particular was powerfully prognostic of incident HF hospitalization and cardiovascular death [28]. However, the mechanisms by which these fairly subtle abnormalities of LV systolic function may predispose to adverse outcomes are unclear.

In addition, we found that patients with HFpEF had reduced mechanical reserve when measured by GLS (increase in GLS < 2%) compared to patients with other causes of dyspnea. Group A and group B did not differ in LVCR when measured by changes in LVEF and force.

Furthermore, multivariable logistic regression analysis confirmed the role of GLS in predicting the diagnosis of HFpEF in patients with dyspnea. Therefore, mechanical reserve measured by GLS could provide adjunctive information during exercise stress echo to identify patients with HFpEF and an inconclusive rest echocardiogram.

Other studies demonstrated reduced contractile reserve when expressed as changes in LVEF or elastance at peak stress but not as mechanical reserve in patients with HFpEF.

Norman et al. demonstrated that during dobutamine stress echo, patients with HFpEF had an impaired contractile response to adrenergic stimulation. Contractile reserve was measured as a change in LVEF, and it was decreased in HFpEF participants compared with control subjects. Borlaug et al. [29] showed that the increase in contractility (assessed by the peak power index, single-beat end-systolic elastance and single-beat preload recruitable stroke work) was 65–85% lower in HFpEF compared with hypertensive and normal controls [30]. Thus, impairments in cardiac reserve, which can be evoked with exercise testing, are well recognized in HFpEF and can be used to detect abnormalities in patients with normal resting-state measures [31].

Therefore, our study highlights the significant alteration of mechanical reserve in patients with HFpEF and the need to assess left ventricular GLS during stress echo to differentiate patients with HFpEF from patients with other causes of dyspnea.

We know that the applicability of deformation imaging during stress echo could have some limitations, mainly due to a high heart rate and increased heart movement. The feasibility and reproducibility of strain analysis during stress echo were previously demonstrated [32]. Good image quality and meticulous acquisition with a dedicated protocol may allow greater applicability of strain analysis during stress echo, as also demonstrated by our variability analysis.

In addition, the measurement of GLS does not significantly increase the duration of the study, with only a slight increase in the post-processing analysis time, and it can have prognostic value.

The measurement of force is simple during stress echo, since it requires only end-systolic volume (ESV) and systolic blood pressure. In contrast to LVEF, force is not dependent on heart rate, preload or afterload changes [33]. The robust pathophysiological basis translates into better prognostic performance when compared to LVEF in patients undergoing stress echo [34,35]. In our study, we did not find significant differences in the force stress/force rest ratio between the two groups, although a higher percentage of patients with HFpEF had a reduced force peak/force rest ratio.

## 6. Conclusions

In our study, reduced LVCR, specifically when assessed using myocardial deformation indices, was more useful than conventional parameters evaluated during diastolic stress tests in the differential diagnosis of HFpEF from other causes of dyspnea. Therefore, measurement of GLS should be encouraged during stress echo to increase the accuracy of the diagnosis of HFpEF.

## Figures and Tables

**Table 1:** Clinical characteristics of the population.

Variables	General Population (62 pts)	Group A (HFpEF) 26 Patients	Group B (Other Causes of Dyspnea) 36 Patients
**Age (Mean ± SD)**	62 ± 10.6	64.3 ± 8.82	61.2 ± 11.2
**Sex (M/F)**	31/31	11/15	20/16
**BMI (kg/m^2^)**	27 ± 3	25.8 ± 3.50	26.8 ± 3.88
**Arterial Hypertension**	51(83%)	22 (42.3%)	17 (32%)
**Diabetes**	23 (38%)	7 (10.7%)	7 (10%)
**Smoking**	12 (20%)	2 (7.6%)	9 (25%)
**Dyslipidemia**	31 (51%)	11 (34.3%)	11 (34.3%)
**ACEI or ARB**	42 (69%)	17 (65%)	24 (66.6%)
**Beta-blockers**	29 (48%)	10 (38.3%)	20 (55.6%)
**Mineralcorticoids**	3 (5%)	1 (5.6%)	2 (5.6%)
**Diuretics**	15 (25%)	6 (33.3%)	8 (22.2%)

Abbreviations. BMI: body mass index. ACEI: angiotensin-converting enzyme inhibitor. ARB: angiotensin receptor blocker. HFpEF: heart failure with preserved left ventricular ejection fraction.

**Table 2:** Echocardiographic changes between rest and stress in group A and group B.

	Group A—HFpEF (26 pts)			Group B—Other Causes of Dyspnea (36 pts)		
	Rest	Stress	*p* Value	Rest	Stress	*p* Value
**LVEF (%)**	58.6 ± 7.95	62.6 ± 6.18	**0.04**	60.8 ± 6.95	64.2 ± 7.35	**0.04**
**GLS (%)**	−17.7 ± 3.56	−18.4 ± 5.62	0.58	−19.1 ± 3.16	−20.9 ± 4.43	**0.04**
**LAVi (mL/m^2^)**	46.5 ± 23.7	51.2 ± 23.9	0.48	35.3 ± 16.4	39.1 ± 21	0.39
**SPAP** **(mmHg)**	23.1 ± 9.24	45.1 ± 10.9	**<0.0001**	16.9 ± 9.72	28.2 ± 11.3	**<0.0001**
**E/e’**	10.7 ± 3.49	15.1 ± 3.89	**0.0001**	8.24 ± 2.36	9.09 ± 4	0.27
**Force (mmHg/mL)**	7.3 ± 2.73	10.9 ± 4.71	**0.0014**	7.85 ± 3.84	12.6 ± 5.16	**<0.0001**
**HR (bpm)**	69 ± 13.6	129 ± 12.6	**<0.0001**	71 ± 8	122 ± 17	**<0.0001**
**SAP (mmHg)**	138 ± 15	190 ± 16	**<0.0001**	140 ± 15	200 ± 30	**<0.0001**
**DAP (mmHg)**	76.8 ± 9	93 ± 14	**<0.0001**	78 ± 11.4	86 ± 13	**0.007**
**LVEDV (mL)**	93 ± 23.61	90 ± 26.16	0.66	90 ± 20	86 ± 13	0.31
**LVESV (mL)**	38 ± 13.9	33 ± 14.5	0.21	37 ± 10	35 ± 9	0.37

Abbreviations. LVEF: left ventricular ejection fraction. GLS: global longitudinal strain. LAVi: left atrial volume indexed. SPAP: systolic pulmonary artery pressure. HR: heart rate. SAP: systolic arterial pressure. DAP: diastolic arterial pressure. LVEDV: left ventricular end-diastolic volume. LVESV: left ventricular end-systolic volume.

**Table 3:** Preserved left ventricular contractile reserve (LVCR) in groups A and B.

	Group A—HFpEF (26 pts)	Group B—Other Causes of Dyspnea (36 pts)	
	STRESS (% pts)	STRESS (% pts)	*p* Value
Changes in GLS ≥ 2	0 (0%)	5 (14%)	**0.04**
Changes in LVEF ≥ 5%	11 (46%)	19 (53%)	0.5
Force peak/force rest > 2	4 (19%)	15 (42%)	0.06

Abbreviations. LVEF: left ventricular ejection fraction. GLS: global longitudinal strain.

**Table 4:** Multivariable logistic regression analysis. Predictors of HFpEF.

Variables	Odds Ratio	95% C.I. Odds Ratio		*p* Value
		Lower	Upper	
**GLS (%) rest**	1.297	1.025	1.643	**0.031**
**GLS (%) peak**	1.396	1.050	1.600	**0.016**
**Δ (peak-rest) GLS (%)**	1.295	1.025	1.636	**0.030**
**LVEF (%) rest**	0.971	0.899	1.049	0.457
**LVEF (%) peak**	0.981	0.880	1.093	0.731
**Δ (peak-rest) LVEF (%)**	1.032	0.931	1.078	0.191
**Force (mmHg/mL) rest**	1.068	0.803	1.422	0.650
**Force (mmHg/mL) peak**	0.976	0.800	1.192	0.531
**Δ Force (mmHg/mL)**	0.921	0.711	1.029	0.089

Abbreviations. LVEF: left ventricular ejection fraction. GLS: global longitudinal strain.

## Data Availability

All raw data will be available upon request to the corresponding author.
